# Protein antigen of bird-related hypersensitivity pneumonitis in pigeon serum and dropping

**DOI:** 10.1186/s12931-017-0555-4

**Published:** 2017-04-20

**Authors:** Tsuyoshi Shirai, Haruhiko Furusawa, Asuka Furukawa, Yuki Ishige, Keisuke Uchida, Yasunari Miyazaki, Yoshinobu Eishi, Naohiko Inase

**Affiliations:** 1 Department of Respiratory Medicine, Tokyo Medical and Dental University, 1-5-45 Yushima, Bunkyo-ku, Tokyo, Japan; 2 Department of Human Pathology, Tokyo Medical and Dental University, Tokyo, Japan; 3 Division of Surgical Pathology, Tokyo Medical and Dental University, Tokyo, Japan

**Keywords:** Hypersensitivity pneumonitis, Extrinsic allergic alveolitis, Bird, Pigeon, Antigen, Protein of immunoglobulin-superfamily

## Abstract

**Background:**

Avian antigen is a common cause of hypersensitivity pneumonitis (HP). Inhalation challenge with pigeon serum and pigeon dropping extract (PDE) elicits a hypersensitivity reaction in patients with bird-related hypersensitivity pneumonitis (BRHP), but the antigenic components in these materials have yet to be fully elucidated.

**Method:**

Pigeon serum, pigeon intestine homogenates, and PDE were immunoblotted with serum samples from 8 patients with BRHP, 2 patients with summer-type HP, 2 patients with humidifier lung, and 3 healthy volunteers. Among the protein spots found in both pigeon serum and PDE, those that reacted with sera from BRHP patients were identified by mass spectrometry. Immunoassays using recombinant protein were performed to confirm the antigenicity of the identified protein. Cytokine production by peripheral blood mononuclear cells (PBMCs) stimulated with recombinant protein was also assessed.

**Results:**

Immunoglobulin lambda-like polypeptide-1 (IGLL-1) was identified from all spots on 2-DE immunoblots of both pigeon serum and PDE. The BRHP patients exhibited higher levels of serum IgG antibody against the recombinant IGLL-1 (rIGLL-1) compared to the control subjects, as well as a stronger PBMCs proliferative response to rIGLL-1. Cytokine production by PBMCs from BRHP patients after rIGLL-1 exposure indicated that the protein could induce Th1 prone immune responses: an increase in TNF-α and an absence of elevated IL-10 production.

**Conclusions:**

Pigeon IGLL-1 was identified as the BRHP antigen present in both pigeon serum and PDE.

**Electronic supplementary material:**

The online version of this article (doi:10.1186/s12931-017-0555-4) contains supplementary material, which is available to authorized users.

## Background

Hypersensitivity pneumonitis (HP) is an allergic lung disease caused by repeated inhalation of causative antigens. Various organic and inorganic particles in home and workplace environments can cause HP [1]. HP is classified into acute and chronic forms based on its clinical features [2]. Chronic HP is characterized by insidiously progressive pulmonary fibrosis with poor prognosis and extremely variable clinical manifestations. Many patients with chronic HP are unaware of exposure to an offending antigen.

Many cases of HP are induced by avian antigen. Reed et al. reported the first avian-associated case of HP in a pigeon breeder, in 1965 [3]. Initially the disease was thought to appear mainly in bird breeders. In the ensuing decades since, the disease has been clearly linked to indirect exposure to avian antigens such as pigeons bred by neighbors, flocks of pigeons in parks, and feather duvets. The disease, therefore, is collectively referred to as “bird-related hypersensitivity pneumonitis” (BRHP). BRHP accounted for 134 (60%) of 222 cases of chronic HP in a Japan-wide epidemiological survey from 2000 to 2009 [4]. In a report from Morell et al., meanwhile, 13 of 46 patients diagnosed with idiopathic pulmonary fibrosis (IPF) were afflicted with BRHP [5]. Patients with BRHP seem to be underestimated in current clinical practice.

Precipitating antibody against a wide range of materials from birds such as droppings, feathers, and serum proteins [6, 7] can be found in the serum of BRHP patients. Yet the antibodies are found in only 35% of patients with chronic BRHP that develops insidiously [8]. The presence or absence of specific antibodies against avian antigen may therefore be insufficient to determine a diagnosis of BRHP.

Natural or provoked inhalation challenge is the most reliable diagnostic procedure for HP. Pigeon dropping extract (PDE) and pigeon serum are likewise used as antigens for inhalation provocation testing for BRHP, and antigen-specific responses have been observed in BRHP patients [9, 10]. As of this writing, however, the lack of a standardized antigen and the risk of disease exacerbation by the provocation challenge have limited the practice of inhalation provocation testing for BRHP to all but a few experienced centers. The diagnosis of chronic BRHP is sometimes left unconfirmed as a consequence. This could be remedied by an accurate identification of the causative antigen from the miscellaneous materials from bird sources associated with the disease.

In the present study we performed an immunoblot analysis of sera from patients with BRHP and identified patient-specific proteins found in both pigeon serum and PDE by mass spectrometry. We then went on to confirm the antigenicity of the identified proteins using recombinant protein by ELISA and peripheral blood mononuclear cells (PBMCs) proliferative assay. In the final part of the study we evaluated the relative changes of cytokine production by PBMCs after stimulation with the recombinant protein.

## Methods

### Subjects

All of the recruited patients were diagnosed at Tokyo Medical and Dental University Hospital from 1998 to 2014. The diagnoses of acute BRHP and chronic BRHP were respectively based on the criteria proposed by Schuyler et al. [11] and Yoshizawa et al. [12].

### Materials

Pigeon serum was collected from veins under the wings and cardiac punctures of five pigeons and pooled. Pigeon intestine and its contents were collected and homogenized in PBS. Serum albumin was depleted by dye affinity chromatography. Dried pigeon droppings were purchased (Greer, Lenoir, NC). PDE was prepared as previously described [13].

### SDS-PAGE and immunoblotting

The proteins from pigeon serum, intestine homogenates, and PDE (7.5 μg each) were separated by SDS-polyacrylamide gel electrophoresis (PAGE) with 10% acrylamide gel and transferred to nitrocellulose membranes. Sera from patients with BRHP and control subjects were used as the primary antibody at a 1:1000 dilution for 30 min at room temperature (RT). Biotin-conjugated goat anti-human IgG (Invitrogen, Carlsbad, CA) was used as the secondary antibody at a 1:500 dilution for 30 min at RT. Blots were visualized using 1:3000 streptoavidin-Cy3 (Sigma Aldrich, St. Louis, MO). Quantity One software (Bio-Rad, Richmond, CA) was used to prepare the images and calculate the molecular weights.

### 2-DE and protein identification by mass spectrometry

Isoelectric focusing/SDS-PAGE was performed with 7 cm IPG strips (Bio-Rad, Richmond, CA) at pH 3–10 using a Protean IEF Cell (Bio-Rad, Richmond, CA) according to the manufacturer’s instructions. The protein spots were visualized with SYPRO Ruby (Lonza Rockland, ME). The proteins were identified by liner ion trap mass spectrometry (FT-MS/MS, Thermo Scientific, Pittsburgh, PA).

### Preparation of recombinant protein

A full-length IGLL-1 cDNA was subcloned into the pHUE vector. The histidine 6-tagged ubiquitin fusion recombinant protein was synthesized in Escherichia coli and purified on Ni-NTA agarose. The ubiquitin was removed with a deubiquitylating enzyme. For the in vitro stimulation of PBMCs, endotoxin-free (<1 Eu/μg) histidine 6-tagged recombinant IGLL-1 (rIGLL-1) was synthesized by GenScript Corp (Piscataway, NJ).

### PDE and recombinant IGLL-1 ELISA

Ten μg/ml of rIGLL-1 and 1 μg (wet weight)/ml of PDE in 15 mM Na_2_CO_3_ or 35 mM NaHCO_3_ buffer (pH 9.5) were coated onto 96-well polystyrene microtiter plates (Thermo Scientific, Pittsburgh, PA) and left overnight at 4 °C. After blocking with 0.5%BSA + PBS-T for 1 h at RT, serum samples at 1:400 dilutions were added to the wells and incubated overnight at 4 °C. A 1:8000 dilution of HRP-conjugated goat anti-human IgG (AbD Serotec Ltd, Oxford, UK) was added together with 0.5%BSA + PBS-T and incubated at 37 °C for 1 h. O-phenylenediamine (OPD) substrate with 0.003% H_2_O_2_ was added and incubated at 37 °C for 30 min in the dark. The reaction was stopped with 2 M H_2_SO_4_ and the optical density at 490 nm was measured with a microplate reader (Bio-Rad, Richmond, CA).

### PBMCs proliferation assay

PBMCs were isolated from the whole blood by Ficoll-Conray density gradient centrifugation. After re-suspending the cells to 2 × 10^6^ cells/ml with RPMI1640 medium and plating them onto a 96-well microplate at 2 × 10^5^ cells/well, the cells were cultured in the presence or absence of 10 μg/ml rIGLL-1 for 72 h in humidified air at 37 °C with 5% CO_2._ After adding ^3^H-thymidine and incubating for an additional 18 h, the cells were harvested onto a filter plate and monitored with a scintillation counter to measure the radioactivity.

### Multiplex cytokine assay

A culture supernatant of PBMCs stimulated with rIGLL-1 was collected on day 5 and examined with a BioPlex human cytokine Th1/Th2 9-plex panel (Bio-Rad, Richmond, CA) to measure the concentrations of human IL-2, IL-4, IL-5, IL-10, IL-12p70, IL-13, GM-CSF, IFN-γ, and TNF-α. Assays were performed on a Luminex 200 system with xPONENT 3.1 software (Luminex Corp., Austin, TX).

### Statistical analysis

All values were analyzed using GraphPad Prism ver 5.0 (GraphPad Software, San Diego, CA). The Mann-Whitney test was used to compare the ELISA values and changes in PBMCs cytokine production between the groups. The frequencies of positive test results in the PBMCs proliferation assay were evaluated by Fisher’s exact test. Spearman’s rank-correlation coefficient and Pearson’s coefficient of correlation were used to measure the correlations between serum IgG levels against PDE and rIGLL-1. A p-value of less than 0.05 was considered statistically significant.

## Results

### Immunoblotting with pigeon serum, PDE, and pigeon intestine

**Fig. 1:**
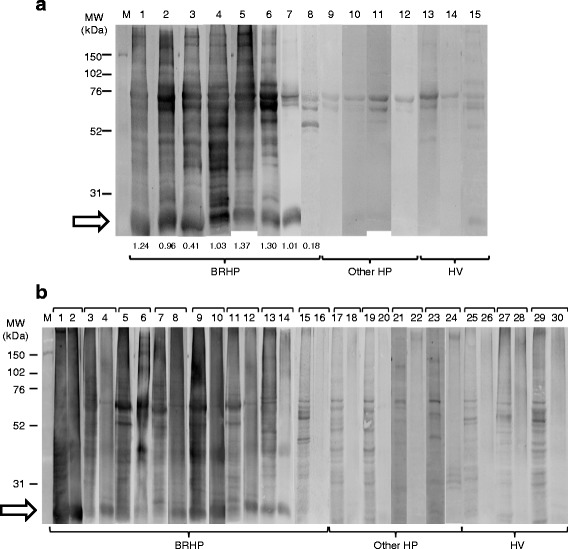
Immunoblotting of pigeon serum (**a**), pigeon intestine homogenates (**b**, odd number), and pigeon dropping extract (PDE) (**b**, even number) with serum samples from patients with bird-related hypersensitivity pneumonitis (BRHP) (**a**; lanes 1–8, **b**; lanes 1–16), patients with other hypersensitivity pneumonitis (HP) (**a**; lanes 9–12, **b**; lanes 17–24), and healthy volunteers (HV) (**a**; lanes 13–15, **b**; lanes 25–30). The arrows indicate the 26 kDa bands observed in all three sample types exposed to sera from 7 out of 8 BRHP patients (Fig. 1**a**, lanes 1–7, Fig. 1**b** lanes 1–14). The numbers indicated under each lane of Fig. 1**a** represent the optical density of serum IgG antibodies against recombinant IGLL-1 in BRHP patients. M, Molecular weight markers

Sera from 8 patients with BRHP (2 acute, 6 chronic), 2 with summer-type HP (SHP), 2 with humidifier lung (HL), and 3 healthy volunteers (HV) were screened by 1-DE immunoblotting of pigeon serum (Fig. 1a), pigeon intestine homogenate and PDE (Fig. 1b). Among the samples exposed to sera from BRHP, multiple immunoreactive bands at various molecular weights were observed in pigeon serum and pigeon intestine homogenate, whereas several bands smaller than 60 kDa were observed in PDE. Among the samples exposed to sera from other HP patients and HVs, multiple bands were observed in pigeon intestine homogenate, only a few bands in the region of 60–70 kDa were observed in pigeon serum, and no bands were observed in PDE. The 26 kDa band was commonly and specifically observed in pigeon serum, pigeon intestine homogenate, and PDE exposed to sera from 7 out of 8 BRHP patients.

### Identification of the 26 kDa protein by mass spectrometry

**Fig. 2:**
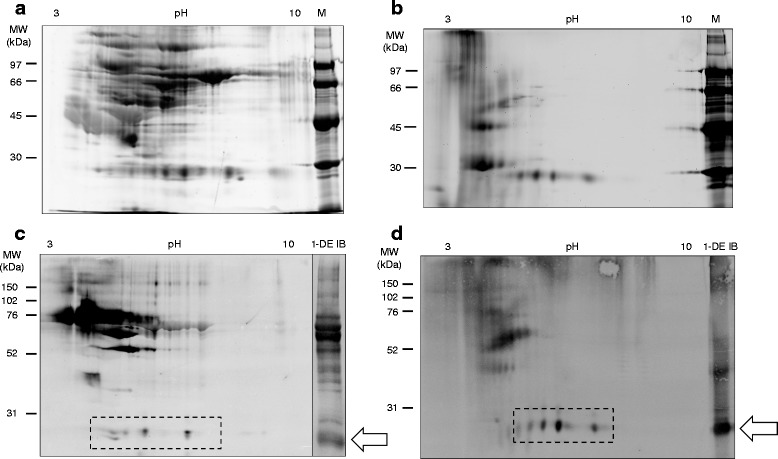
Proteins from pigeon serum (**a**) and pigeon dropping extract (PDE) (**b**) were separated by 2-DE and visualized by SYPRO Ruby staining. Representative results of 2-DE immunoblotting of pigeon serum (**c**) and PDE (**d**) using sera from BRHP patients are shown. The 1-DE immunoblot of each sample is shown in the right panel for comparison. Proteins in the 26 kDa band observed on 1-DE immunoblotting (*indicated by arrow*) were separated into several protein spots (*indicated within broken-lined boxes*). Sera from BRHP patients reacted with 4 spots from the proteins from pigeon serum and 5 spots from the PDE in a similar pH range. M, Molecular weight markers; 1-DE IB, 1-DE immunoblot

**Fig. 3:**
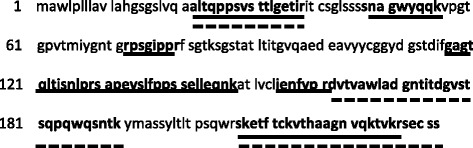
Amino acid sequence of IGLL-1 (232 amino acids). The amino acid residues in bold represent the peptides identified by mass spectrometry of pigeon serum (*solid line*) and pigeon dropping extract (PDE) (*dashed line*) with 41 and 40% coverage, respectively

To separate the proteins in the 26 kDa band observed on the 1-DE immunoblot of pigeon serum and PDE, we performed 2-DE and visualized the proteins by staining with SYPRO Ruby stain (Fig. 2a, b). Of the protein spots separated by 2-DE, 4 spots of pigeon serum and 5 spots of PDE were reactive to the sera from the BRHP patients in the region of 26 kDa (Fig. 2c, d). None of the sera from the HVs or other HP patients reacted to any of these protein spots (data not shown). The corresponding spots observed in pigeon serum and PDE were excised from gels and analyzed by liner ion trap FT-MS/MS mass spectrometry. Immunoglobulin-lambda-like polypeptide-1 (IGLL-1) was identified with high confidence (20 and 13 unique peptide were matched from pigeon serum and PDE, respectively) from all of excised spots of pigeon serum and PDE protein (Fig. 3).

### The levels of anti IGLL-1 antibody in patients with BRHP

**Fig. 4:**
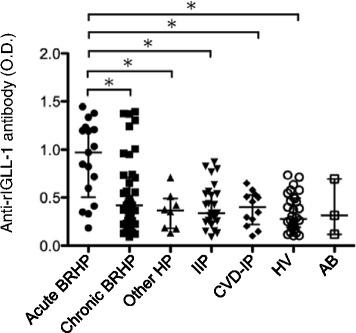
Optical density (O.D.) of serum IgG antibodies against recombinant IGLL-1 (rIGLL-1) were measured by ELISA. Serum samples were obtained from 17 patients with acute bird-related hypersensitivity pneumonitis (BRHP), 42 with chronic BRHP, 8 with other hypersensitivity pneumonitis (HP), 26 with idiopathic interstitial pneumonia (IIP), 12 with collagen vascular disease-associated interstitial pneumonitis (CVD-IP), 30 healthy volunteers (HVs), and 3 asymptomatic breeders (ABs). Results represent median ± IQRs. * *p* < 0.05

To confirm the antigenicity of the identified protein, we evaluated serum samples obtained from 17 patients with acute BRHP, 42 with chronic BRHP, 8 with other HP (4 with SHP, 3 with HL, 1 with chronic HP induced by wheat flour), 26 with idiopathic interstitial pneumonia (IIP), 12 with collagen vascular disease-associated interstitial pneumonitis (CVD-IP), 30 HVs, and 3 asymptomatic breeders (AB) (Fig. 4). The clinical characteristics of the subjects are presented in Table 1. The detailed characteristics of the patients with acute and chronic BRHP are presented in Table 2.

**Table 1:** Characteristics of subject groups

	BRHP	Other HP	IIP	CVD-IP	HV	AB
	(*n* = 59)	(*n* = 8)	(*n* = 26)	(*n* = 12)	(*n* = 30)	(*n* = 3)
Age, years	62 (31–70)	60 (57–68)	68 (64–73)	62 (60–67)	37 (33–46)	45 (37–48)
Sex, male/female	32/27	8/0	21/5	7/5	24/6	1/2
NS/ES/CS, %	57.6/35.6/5.1	12.5/87.5/0	26.9/53.8/15.4	33.3/58.3/8.3	100/0/0	100/0/0
FVC, % predicted	72.6 (59.2–91.3)	76.0 (65.6–93.1)	72.2 (65.8–81.6)	74.5 (68.9–88.6)	NA	NA
BAL cell counts Lymphocytes, %	53.5 (29.7–83.0)	40.2 (30.8–68.0)	6.9 (4.5–10.7)	11.2 (5.9–14.8)	NA	NA
Serum KL-6, U/ml	1419 (742–3222)	1417 (1066–1620)	1151 (903–1356)	1370 (1093–1676)	NA	NA

Data are presented as median values and interquartile ranges unless otherwise indicated

*AB* asymptomatic breeder, *BAL* bronchoalveolar lavage, *BRHP* bird related hypersensitivity pneumonitis, *CVD-IP* collagen vascular disease-associated interstitial pneumonia, *FVC* forced expiratory capacity, *HP* hypersensitivity pneumonitis, *HV* healthy volunteers, *IIP* idiopathic interstitial pneumonia, *NA* not applicable, *NS/ES/CS* never-smokers/ex-smokers/current-smokers

**Table 2:** Characteristics of acute and chronic BRHP Patients

	Acute BRHP	Chronic BRHP
	(*n* = 17)	(*n* = 42)
Age, yr	57 (48–64)	64 (56–71)
Sex, male/female	6/11	26/16
Breed history of birds N/F/C, %	0/5.9/94.1	35.7/33.3/31.0†
Exposure periods, mo	90 (30–153)	120 (63–216)
Kind of birds C/G/Pa/Ps, %	44.4/0/5.6/50	42.9/10.7/10.7/35.7
BAL cell counts Lymphocytes, %	81.8 (63.8–90.0)	42.0 (6.9–61.8)*
Serum KL-6, U/ml	2606 (1000–5766)	1275 (724–2595)

Data are presented as median values and interquartile ranges unless otherwise indicated

*BAL* bronchoalveolar lavage, *N/F/C* non-breeders/former-breeders/current-breeders, *C/G/Pa/Ps* Columbiformes/Galliformes/Passeriformes/Psitacciformes

*p < 0.05 compared with Acute BRHP. †*p* < 0.01 compared with Acute BRHP

The serum IgG levels against rIGLL-1 were significantly higher (*p* < 0.05) in acute BRHP patients than in chronic BRHP patients, IIP patients, and HVs. The chronic BRHP patients tended to have higher levels than the HVs. There was a strong positive correlation (*r* = 0.79, *p* < 0.01) between the levels of serum IgG antibodies against rIGLL-1 and PDE (Additional file 1: Figure S1).

### PBMCs proliferation assay

Antigen-specific T-cell proliferation was analyzed by stimulating PBMCs from 5 patients with acute BRHP, 17 with chronic BRHP, 4 with other HP (2 with SHP, 2 with home-related HP), 8 with other fibrotic lung diseases (FLDs), 10 HVs, and 2 ABs with LPS-free rIGLL-1 and then measuring the cell proliferation according to the uptake of ^3^H-thymidine. The concentration of rIGLL-1 as a stimulus was 10 μg/ml. The PBMCs from 2 out of 5 (40%) patients with acute BRHP and 7 out of 17 (41.2%) patients with chronic BRHP were positive (SI > 2). No positive results were observed in the PBMCs from other HP (*n* = 4), the other FLD patients (*n* = 8), or the HVs (*n* = 10). Few Abs (*n* = 2) were used for the assay, but the PBMCs obtained from them showed a high SI.

### Cytokine production from stimulated PBMCs

We investigated whether the antigen-stimulation influenced the cytokine profiles by analyzing the production of IL-2, IL-4, IL-5, IL-10, IL-12p70, IL-13, TNF-α, and IFN-γ by PBMCs from 14 BRHP patients (4 acute, 10 chronic) and 6 HVs stimulated with rIGLL-1. The protein levels of IL-10 after the rIGLL-1 stimulation were significantly increased in both the BRHP patients and HVs. TNF-α was significantly decreased in the HVs, but tended to increase in most of the BRHP patients. No consistent pattern of increase or decrease was observed in the other cytokines (Additional file 2: Figure S2).

**Fig. 5:**
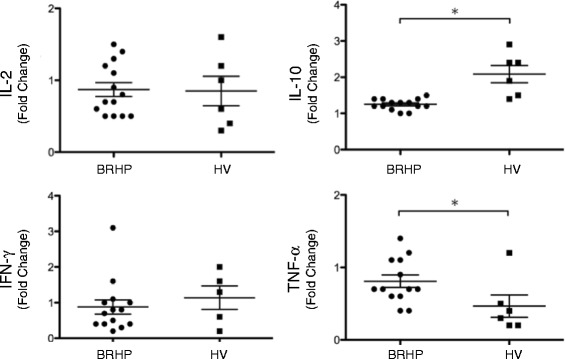
Fold changes in cytokine production by PBMCs from 14 patients with bird-related hypersensitivity pneumonitis (BRHP) and 6 healthy volunteers (HV) stimulated with recombinant IGLL-1. Graphs are represented as mean ± SEM. * *p* < 0.05

Fold changes in cytokine concentrations between baseline and post-stimulation were compared between the BRHP patients and HVs. IL-10 was significantly lower and TNF-α was significantly higher in BRHP patients than in HVs (Fig. 5). The changes in IL-2 and IFN-γ did not significantly differ between the groups.

## Discussion

In the present study we identified IGLL-1 as the antigenic protein of BRHP using immunoblot analysis and mass spectrometry. The serum levels of anti-IGLL-1 antibody were elevated in a disease-specific manner, and rIGLL-1 stimulated PBMCs proliferation in approximately 40% of the patients.

Researchers investigating the causative antigen of BRHP have reported several important findings. In 1965, Barboriak et al. reported that sera from BRHP patients and ABs showed a specific reaction to pigeon sera [6]. Among these serum proteins, antibodies against pigeon immunoglobulins are quantitatively higher in patients with BRHP than in Abs [14]. Pigeon IgA, the major antigenic component present in both PDE and feather bloom [15–18], may also induce a T-cell response in PBMCs obtained from BRHP patients [19]. Based on these results, Mcsharry et al. speculated that pigeon immunoglobulin light chain may be a common antigenic component of different classes of gamma-globulin proteins commonly present in pigeon serum, droppings, and bloom [20]. The studies done so far have generally used crude or fractionated pigeon serum or extracts of bloom or droppings. Few studies have demonstrated the antigenicity of pigeon immunoglobulins by isolating and identifying antigens from miscellaneous materials. In the present study, we identified IGLL-1 as the candidate protein by using mass spectrometry. We could also confirm the disease-specific antigenicity in sera from patients with BRHP. HP pathogenesis is generally characterized by an inflammation of the lung parenchyma that can progress to lung fibrosis involving both humoral and T-cell-mediated responses [21]. Our ELISA (humoral) and PBMCs proliferation testing (cell-mediate) in this study confirmed that IGLL-1 provokes disease-specific responses.

IGLL-1, alternatively called λ5, is an invariant polypeptide belonging to the immunoglobulin super family. IGLL-1 composes the surrogate light chain (SLC) of the pre-B-cell receptor with VpreB, which is critical for B cell development in the bone marrow. The protein is composed of 232 amino acid residues divided into two domains: an immunoglobulin-like domain (Ig-like) consisting of a unique tail of SLC [22, 23] and an immunoglobulin-constant domain (Ig-C) consisting of a basic structure of immunoglobulin molecules. The protein is produced in excess of heavy chain and is secreted into the extracellular environment, where it becomes detectable in serum, skin, the digestive tract, and urine in humans.

According to the BLAST database, the amino acid sequence of pigeon IGLL-1 (Accession no. XP_005503923.1) and immunoglobulin light chains and IGLL of duck, goose, chicken, and parakeet has a 58–66% sequence identity, whereas that of mammals has a slightly lower sequence identity of 56%. Of note, several amino acid residues highly conserved only among birds were observed in the N-terminal domain (Additional file 3: Figure S3). This specificity to birds may explain the cross-reactivity of patient sera to other unexposed avian species [24]. Our result is therefore consistent with the speculation from Mcsharry et al. that immunoglobulin light chain may cause the BRHP.

We also performed multiplex cytokine assays using culture supernatant of PBMCs stimulated with rIGLL-1. The assays revealed increased production of TNF-α and reduced production of IL-10. In earlier studies we found that IL-10 may have a protective effect against the pathogenesis of isocyanate-induced HP [25]. Pro-inflammatory cytokines were enhanced in PBMCs from HP patients, whereas normal volunteers with current occupational exposure showed elevated levels of mRNA expression of IL-10, suggesting the presence of sensitized cells and protection against disease through enhanced IL-10 production. IL-10-deficient mice exposed to *Saccharopolyspora rectivirgula* exhibited an increase in alveolitis associated with an upregulation of IFN-γ [26]. In a human study of gene expression profiles, the Th1 type chemokine IP-10 appeared to be essential for the recruitment of activated T cells through the chemokine receptor CXCR3 in acute HP [27]. The clinical behaviors of HP are associated with the Th1/Th2 polarization of T-cells. In an earlier study of the relationship between the Th1/Th2 balance and pathology of chronic HP, a shift to a Th2 immune response appeared to play a role in the progression of usual interstitial pneumonia-like lesions, while a shift to a Th1-predominant immune response was seen in the progression of cellular non-specific interstitial pneumonia/organizing-pneumonia-like lesions [28]. Though further study is needed for confirmation, IGLL-1 may play some antigenic role through IL-10 in the pathogenesis of HP.

While only a few ABs were studied, the proliferative response of the rIGLL-1-stimulated PBMCs from the ABs was comparable to the high response from the PBMCs from the BRHP patients. This result corroborates previous studies showing antigen-specific cell-mediated immune response in both patients and Abs [29, 30]. It also indicates that other genetic and environmental factors, such as smoking and impairment of immune tolerance mediated by regulatory T-cells [31], might induce and perpetuate inflammation.

Our study has several limitations. First, not all of the patients with chronic HP included in this study underwent the inhalation provocation challenge. We diagnosed these patients as chronic HP based on a combination of antigen exposure and compatible clinical, immunological, radiological, and pathological findings. Second, many of the protein spots specific to BRHP in the immunoblot analysis were also observed in both PDE and pigeon serum. In this study we analyzed only few of these spots at 26 kDa. While IGLL-1 may not be the only disease-specific antigen, our findings still attest to its usefulness for diagnosing BRHP. Our experiments confirmed the disease-specific antigenicity of IGLL-1 and demonstrated that the protein’s action in provoking Th1 response and inhibiting Th2 response may be specific to BRHP patients. Third, the positive rate of PBMCs proliferation assay stimulated by rIGLL-1 was relatively low, only around 40% of the patients showed positive results. The proliferation of PBMCs depends on numbers of antigen-sensitized T cells in PBMC. However, because these cells are exceedingly rare in the blood, PBMCs proliferation assay is specific but not sufficiently sensitive for a diagnosis. As previously reported, patients with BRHP showed increasing proliferation not more than 50-60% with PBMCs proliferation assay using crude pigeon plasma [32, 33].

## Conclusion

This is the first study identifying the antigenic protein contained in both pigeon serum and dropping by demonstrating the presence of specific antibodies in patients’ sera and an increase in PBMCs proliferation in response to stimulation with recombinant protein. The change of cytokine production by PBMCs after stimulation by recombinant protein was also found to be consistent with the pathogenesis of HP.

## Additional files

Additional file 1: **Figure S1.** Relationship between optical density (O.D.) at 490 nm of serum IgG antibodies against recombinant IGLL-1 (rIGLL-1) and pigeon dropping extract (PDE) (*n* = 59). (PPTX 85 kb)

Additional file 2: **Figure S2.** Production of IL-2, IL-5, IL-10, IL-12p70, IL-13, TNF-α, and IFN-γ cytokines by PBMCs from 14 patients with bird-related hypersensitivity pneumonitis (BRHP) (4 acute BRHP, 10 chronic BRHP) and 6 healthy volunteers (HV). * *p* < 0.05. (PPTX 334 kb)

Additional file 3: **Figure S3.** Amino acid sequence alignments of pigeon IGLL-1, immunoglobulin light chains, and IGLL of other birds and mammalian species. Residues highly conserved across all species are highlighted in grey. Residues conserved only among birds are highlighted in black. The accession numbers of the sequences are as follows: pigeon IGLL-1 (XP_005503923.1), duck Ig lambda chain (S49449), goose immunoglobulin light chain (AEB71783.1), chicken Ig light chain (AAA48859.1), parakeet IGLL-1 (XP_012984154.1), monkey immunoglobulin lambda light chain (ADX62855.1), gorilla IGLL-5 (XP_004063179.1), and human Ig lambda chain (S25744). (PPTX 88 kb)

## Abbreviations

AB: Asymptomatic breeder
BRHP: Bird-related hypersensitivity pneumonitis
CVD-IP: Collagen vascular disease-associated interstitial pneumonitis
FLD: Fibrotic lung disease
HL: Humidifier lung
HP: Hyper sensitivity pneumonitis
HV: Healthy volunteers
IGLL-1: Immunoglobulin lambda-like polypeptide-1
IIP: Idiopathic interstitial pneumonia
IPF: Idiopathic pulmonary fibrosis
PBMC: Peripheral blood mononuclear cell
PDE: Pigeon dropping extract
SHP: Summer-type hypersensitivity pneumonitis
