# Supplementary figures and images for: Protein antigen of bird-related hypersensitivity pneumonitis in pigeon serum and dropping
Source: Respir Res. 2017 Apr 20;18:65. doi: 10.1186/s12931-017-0555-4 (PMC5397797; doi:10.1186/s12931-017-0555-4)

## Slide 1
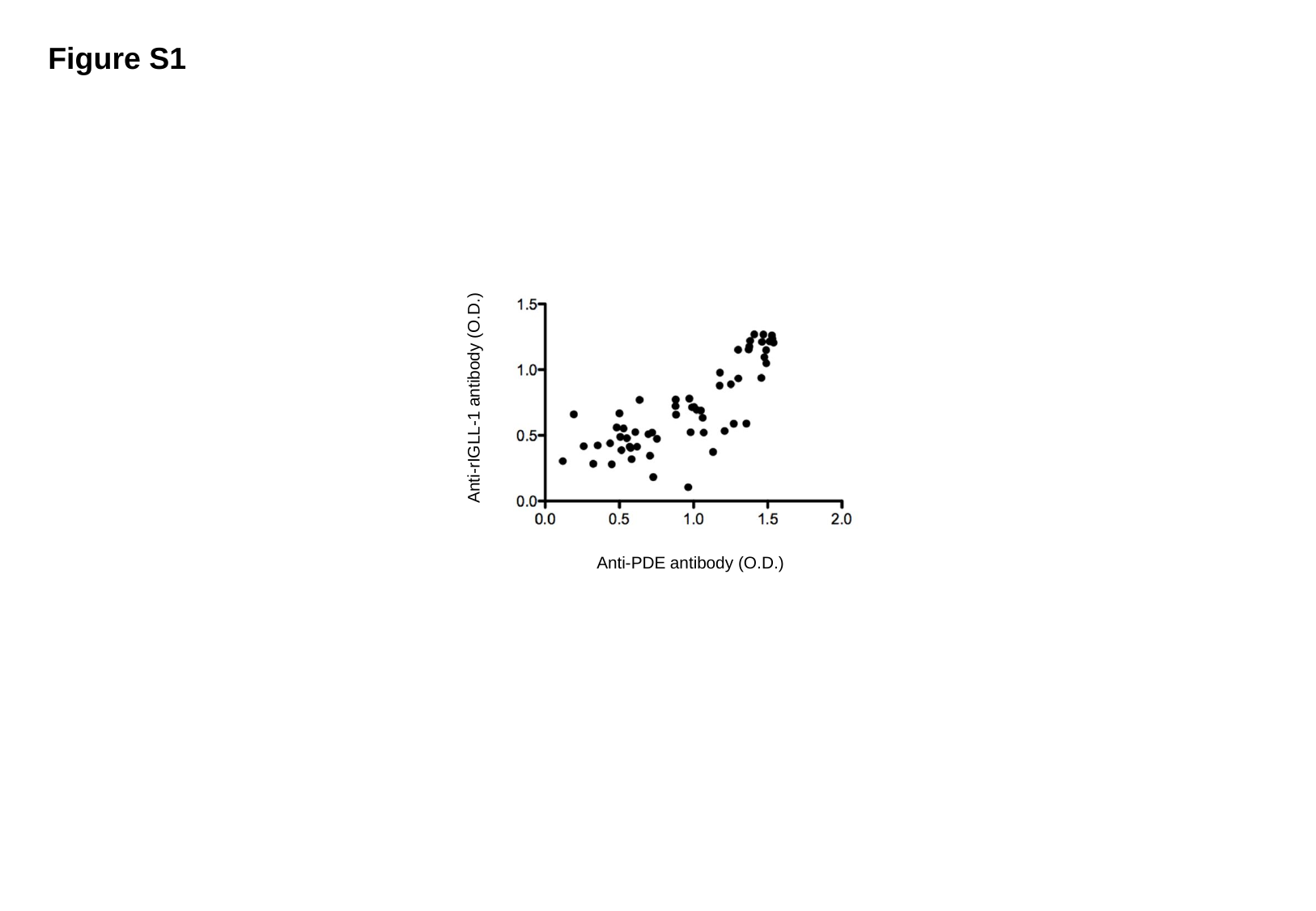

Figure S1
Anti-rIGLL-1 antibody (O.D.)
Anti-PDE antibody (O.D.)

Supplement: Supplementary file 1 — Figure S1. Relationship between optical density (O.D.) at 490 nm of serum IgG antibodies against recombinant IGLL-1 (rIGLL-1) and pigeon dropping extract (PDE) (n = 59). (PPTX 85 kb) [file 12931_2017_555_MOESM1_ESM.pptx]

## Slide 1
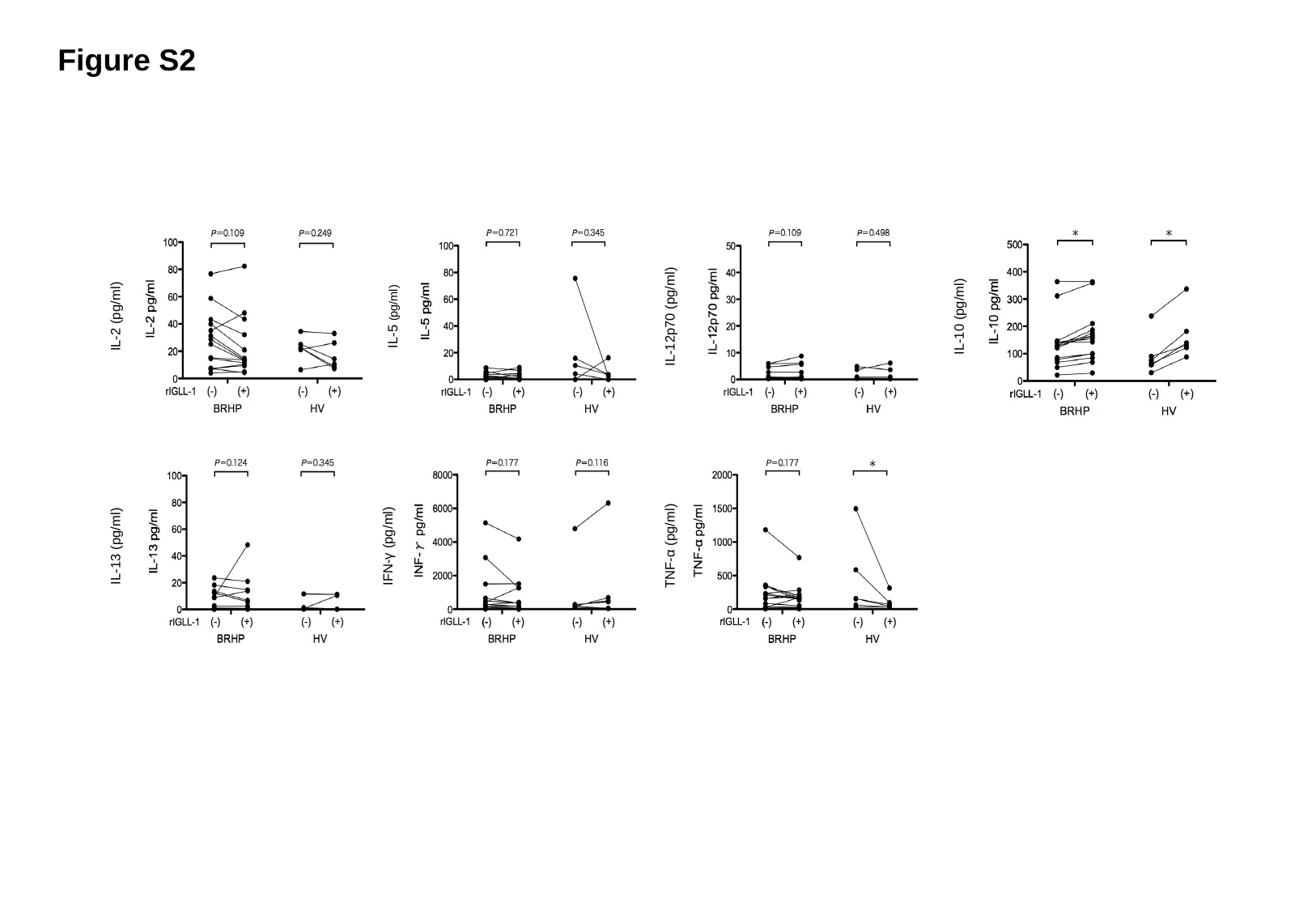

Figure S2
IL-2 (pg/ml)
IL-5 (pg/ml)
IL-12p70 (pg/ml)
IL-10 (pg/ml)
IL-13 (pg/ml)
IFN-γ (pg/ml)
TNF-α (pg/ml)

Supplement: Supplementary file 2 — Figure S2. Production of IL-2, IL-5, IL-10, IL-12p70, IL-13, TNF-α, and IFN-γ cytokines by PBMCs from 14 patients with bird-related hypersensitivity pneumonitis (BRHP) (4 acute BRHP, 10 chronic BRHP) and 6 healthy volunteers (HV). * p < 0.05. (PPTX 334 kb) [file 12931_2017_555_MOESM2_ESM.pptx]
