# Supplementary material for: Protein antigen of bird-related hypersensitivity pneumonitis in pigeon serum and dropping
Source: Respir Res. 2017 Apr 20;18:65. doi: 10.1186/s12931-017-0555-4 (PMC5397797; doi:10.1186/s12931-017-0555-4)
Supplement: Supplementary file 3 — Figure S3. Amino acid sequence alignments of pigeon IGLL-1, immunoglobulin light chains, and IGLL of other birds and mammalian species. Residues highly conserved across all species are highlighted in grey. Residues conserved only among birds are highlighted in black. The accession numbers of the sequences are as follows: pigeon IGLL-1 (XP_005503923.1), duck Ig lambda chain (S49449), goose immunoglobulin light chain (AEB71783.1), chicken Ig light chain (AAA48859.1), parakeet IGLL-1 (XP_012984154.1), monkey immunoglobulin lambda light chain (ADX62855.1), gorilla IGLL-5 (XP_004063179.1), and human Ig lambda chain (S25744). (PPTX 88 kb) [file 12931_2017_555_MOESM3_ESM.pptx]

## Slide 1
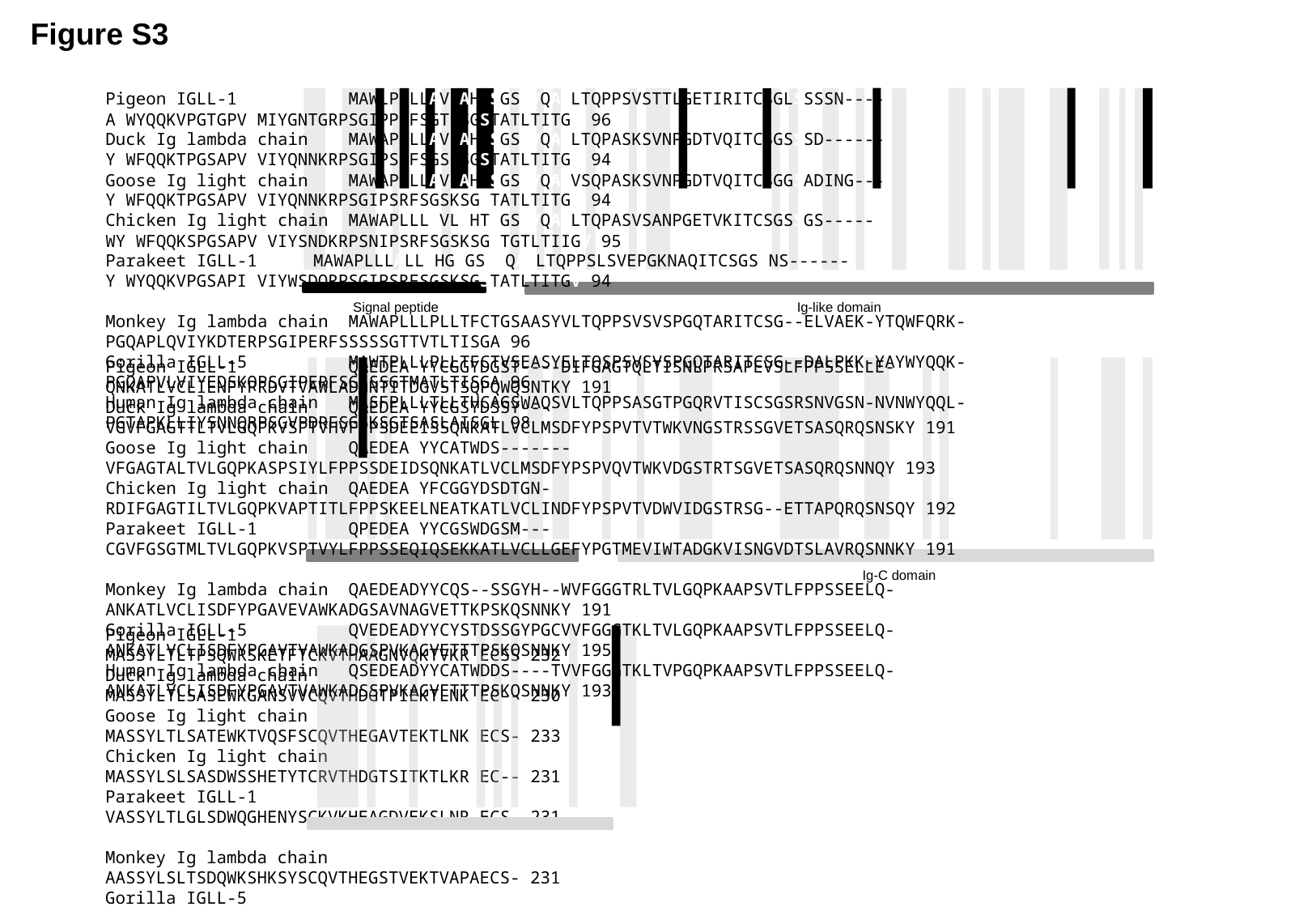

Figure S3
Pigeon IGLL-1 MAWLPLLLAVLAHGSGSLVQAALTQPPSVSTTLGETIRITCSGLSSSSN----AGWYQQKVPGTGPVTMIYGNTGRPSGIPPRFSGTKSGSTATLTITGV 96
Duck Ig lambda chain MAWAPLLLAVLAHTSGSLVQAALTQPASKSVNPGDTVQITCSGSSSD------YGWFQQKTPGSAPVTVIYQNNKRPSGIPSRFSGSKSGSTATLTITGV 94
Goose Ig light chain MAWAPLLLAVLAHTSGSLVQAAVSQPASKSVNPGDTVQITCSGGSADING---YGWFQQKTPGSAPVTVIYQNNKRPSGIPSRFSGSKSGSTATLTITGV 94
Chicken Ig light chain MAWAPLLLAVLAHTSGSLVQAALTQPASVSANPGETVKITCSGSSGS-----WYGWFQQKSPGSAPVTVIYSNDKRPSNIPSRFSGSKSGSTGTLTIIGV 95
Parakeet IGLL-1　 MAWAPLLLALLAHGSGSLVQAALTQPPSLSVEPGKNAQITCSGSSNS------YGWYQQKVPGSAPITVIYWSDQRPSGIPSRFSGSKSGSTATLTITGV 94
Monkey Ig lambda chain MAWAPLLLPLLTFCTGSAASYVLTQPPSVSVSPGQTARITCSG--ELVAEK-YTQWFQRK-PGQAPLQVIYKDTERPSGIPERFSSSSSGTTVTLTISGA 96
Gorilla IGLL-5 MAWTPLLLPLLTFCTVSEASYELTQSPSVSVSPGQTARITCSG--DALPKK-YAYWYQQK-PGQAPVLVIYEDSKQPSGIPERFSGSSSGTMATLTISGA 96
Human Ig lambda chain MASFPLLLTLLTHCAGSWAQSVLTQPPSASGTPGQRVTISCSGSRSNVGSN-NVNWYQQL-PGTAPKLLIYSNNQRPSGVPDRFSGSKSGTSASLAISGL 98
Signal peptide
Ig-like domain
Pigeon IGLL-1 QAEDEAVYYCGGYDGST----DIFGAGTQLTISNLPRSAPEVSLFPPSSELLE-QNKATLVCLIENFYPRDVTVAWLADGNTITDGVSTSQPQWQSNTKY 191
Duck Ig lambda chain QAEDEAVYYCGSYDSSY---VGVFGAGTTLTVLGQPKVSPTVHVFPPSDEEISSQNKATLVCLMSDFYPSPVTVTWKVNGSTRSSGVETSASQRQSNSKY 191
Goose Ig light chain QAEDEAVYYCATWDS-------VFGAGTALTVLGQPKASPSIYLFPPSSDEIDSQNKATLVCLMSDFYPSPVQVTWKVDGSTRTSGVETSASQRQSNNQY 193
Chicken Ig light chain QAEDEAVYFCGGYDSDTGN-RDIFGAGTILTVLGQPKVAPTITLFPPSKEELNEATKATLVCLINDFYPSPVTVDWVIDGSTRSG--ETTAPQRQSNSQY 192
Parakeet IGLL-1 QPEDEAVYYCGSWDGSM---CGVFGSGTMLTVLGQPKVSPTVYLFPPSSEQIQSEKKATLVCLLGEFYPGTMEVIWTADGKVISNGVDTSLAVRQSNNKY 191
Monkey Ig lambda chain QAEDEADYYCQS--SSGYH--WVFGGGTRLTVLGQPKAAPSVTLFPPSSEELQ-ANKATLVCLISDFYPGAVEVAWKADGSAVNAGVETTKPSKQSNNKY 191
Gorilla IGLL-5 QVEDEADYYCYSTDSSGYPGCVVFGGGTKLTVLGQPKAAPSVTLFPPSSEELQ-ANKATLVCLISDFYPGAVTVAWKADGSPVKAGVETTTPSKQSNNKY 195
Human Ig lambda chain QSEDEADYYCATWDDS----TVVFGGGTKLTVPGQPKAAPSVTLFPPSSEELQ-ANKATLVCLISDFYPGAVTVAWKADSSPVKAGVETTTPSKQSNNKY 193
Ig-C domain
Pigeon IGLL-1 MASSYLTLTPSQWRSKETFTCKVTHAAGNVQKTVKRSECSS 232
Duck Ig lambda chain MASSYLTLSASEWKGANSVVCQVTHDGTPIEKTLNKSEC–- 230
Goose Ig light chain MASSYLTLSATEWKTVQSFSCQVTHEGAVTEKTLNKSECS- 233
Chicken Ig light chain MASSYLSLSASDWSSHETYTCRVTHDGTSITKTLKRSEC-- 231
Parakeet IGLL-1 VASSYLTLGLSDWQGHENYSCKVKHEAGDVEKSLNRSECS- 231
Monkey Ig lambda chain AASSYLSLTSDQWKSHKSYSCQVTHEGSTVEKTVAPAECS- 231
Gorilla IGLL-5 AASSYLSLTPEQWKSHKSYSCQVTHEGSTVEKTVAPTECS- 235
Human Ig lambda chain AASSYLSLTPEQWKSHRSYSCQVTHEGSTVEKTVAPTECS- 233
